# Olfactory Neuroblastoma Is Not Always Located at the Roof of the Nasal Cavity

**DOI:** 10.5334/jbsr.3562

**Published:** 2024-04-12

**Authors:** Pornrujee Hirunpat, Theeraphol Panyaping, Siriporn Hirunpat

**Affiliations:** 1 Chakri Naruebodindra Medical Institute, Faculty of Medicine, Ramathibodi Hospital, Mahidol University, Samut Prakan, Thailand; 2 Division of Neurological Radiology, Department of Diagnostic and Therapeutic Radiology, Ramathibodi Hospital, Mahidol University, Bangkok, Thailand; 3 Department of Radiology, Faculty of Medicine, Prince of Songkla University, Hat-Yai, Songkhla, Thailand

**Keywords:** Olfactory neuroblastoma, computed tomography, magnetic resonance imaging, nasal cavity, sinuses, esthesioneuroblastoma

## Abstract

**Objectives:**

To evaluate the imaging characteristics of the tumor, emphasizing its location, and to determine the frequency of typical and atypical locations of olfactory neuroblastoma (ONB).

**Materials and Methods:**

We retrospectively reviewed the computed tomography and magnetic resonance imaging findings of patients with pathologically proven ONB between April 2000 and April 2023. Demographic information, chief complaints, tumor location, and tumor extension were extracted.

**Results:**

Of the 58 patients, 50 (86.2%) had the epicenter of the mass at the superior part of the nasal cavity, while eight patients (13.8%) had the epicenter at other atypical locations: seven patients (12.1%) at the middle part of the nasal cavity and one patient (1.7%) within both sphenoid sinuses.

**Conclusion:**

ONB is not always present in the upper part or the roof of the nasal cavity, and a significant number of ONBs are occasionally found in the rest of the nasal cavity and other atypical locations.

## Introduction

Olfactory neuroblastoma (ONB) or esthesioneuro-blastoma is a rare, aggressive, and malignant neuroectodermal tumor of the nasal cavity. It is thought to arise from the basal neural cells of the olfactory mucosa and accounts for approximately 3% of all sinonasal malignancies [1].

ONB most commonly originates in the superior nasal cavity, especially in the olfactory recess [2], whereas other atypical locations are believed to be extremely rare [3, 4]. A few small sample size studies have reported a 100% frequency of tumor formation in the superior nasal cavity [5, 6]. Radiologists usually include ONB in the differential diagnosis when the epicenter of the tumor is within the superior part of the nasal cavity; however, it is rarely considered when the center of the lesion is in the rest of the nasal cavity or another location in the head and neck region. Nevertheless, ONB in other atypical locations has been reported in recent literature, such as the rest of the nasal cavity, maxillary sinus, ethmoid sinus, sphenoid sinus, pterygopalatine fossa, optic pathways, and sphenoclival region [1, 4, 7–12].

Imaging is critical in gaining an accurate diagnosis, and the tumor site is an essential indicator that directs the correct diagnosis. Studies on the frequency of atypical ONB are scarce, and most studies have reported cases because of its rarity. Therefore, we tried to gather as many patients as possible from the three institutions and evaluate the imaging characteristics of the tumor, emphasizing the tumor location, to determine the frequency of the typical and atypical locations of ONB.

## Materials and Methods

This retrospective multicenter study was approved by the Institutional Review Board, and the requirement for informed consent from the study participants was waived because of the retrospective design of this study.

### Patient Population

Participants were retrospectively identified through the radiologist information systems from three hospital databases for requests and reports via the specific terms “Esthesioneuroblastoma” or “Olfactory Neuroblastoma”: between April 2000 and April 2023. The final diagnoses of patients were reviewed based on their medical records. The inclusion criteria were as follows: (a) pathologically confirmed diagnosis of ONB and (b) complete pretreatment magnetic resonance imaging (MRI) and/or computed tomography (CT). Information was extracted from each patient’s records, including age at the time of imaging, sex, and clinical symptoms.

### Image Acquisition

CT and MRI protocols were not standardized across centers because of their retrospective nature and the different imaging scanning protocols in different institutions. However, essential images in the axial and coronal planes of the entire paranasal sinus, either CT, MRI, or both, must be included for the evaluation of all patients.

### Image Evaluation

Images were retrospectively reviewed by two neuroradiologists simultaneously using the Picture Archiving and Communication System. Both neuroradiologists were not blinded to the pathological diagnosis of the ONB. For participants with multiple CT and MRI scans, only the initial scans were analyzed. Both initial CT and MRI scans were collected if the time between the two studies was less than 3 months. The location of the primary tumor, including the epicenter and side of the nasal cavity, was assessed. We classified the epicenter of the tumor as either predominantly superior, middle, or inferior nasal cavity. The superior nasal cavity extends from the cribriform plate to the superior aspect of the middle turbinate. The middle and inferior nasal cavities are regions below the superior aspect of the middle and inferior nasal turbinates, respectively. Any lesions abutting the roof of the right nasal cavity or the cribriform plate were considered epicenters at the superior part. The paranasal sinuses and the intraorbital and intracranial extensions of the tumor were also recorded.

### Statistical Analysis

No statistical comparisons were performed. Categoric outcomes were reported as numbers (percentage), and continuous outcomes were reported as mean ± standard deviation.

## Results

### Patient Demographics and Clinical Presentation

A total of 58 patients (35 men and 23 women; age range 23 to 84 years; mean age 50.6 ± 15.6 years) with pathologic proven ONB were enrolled in this study. Fifty CT scans and 36 MRI scans were evaluated. The patient demographics and clinical presentations are summarized in Table 1.

**Table 1:** Demographic data and presenting symptoms of patients with olfactory neuroblastoma

	NO. (%)
Number of patients	58 (100)
Age range Mean age (SD) (yr)	23–84 50.61 (±15.6)
Sex Male Female	35 (60.3) 23 (39.7)
Imaging studies CT MRI	50 36
Presenting symptoms Nasal obstruction Epistaxis Anosmia Rhinorrhea Proptosis Eye pain	25 (43.1) 19 (32.8) 5 (8.6) 4 (6.9) 2 (3.4) 3 (5.2)

The location, epicenter, and extension of tumors proven to be ONB are summarized in Table 2.

**Table 2:** Location, epicenter, and extension of olfactory neuroblastoma

LOCATION AND EXTENSION	NO. (%)
Side (nasal cavity) Right Left Both Midline	27 (46.6) 27 (46.6) 3 (5.2) 1 (1.7)
Epicenter Superior Other – Middle – Sphenoid sinus invade sella turcica	50 (86.2) 8 (13.8) 7 (12.1) 1 (1.7)
Extension Frontal sinus Ethmoid sinus Maxillary sinus Sphenoid sinus Intra-orbit Intra-cranium	34 (58.6) 50 (86.2) 24 (41.4) 30 (51.7) 26 (44.8) 44 (75.9)

**Figure 1:**
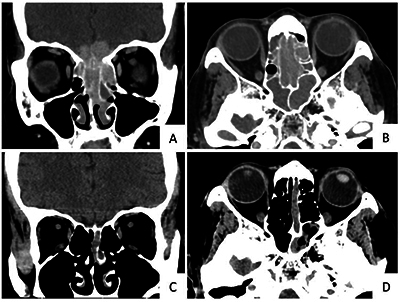
Typical location of olfactory neuroblastoma (ONB), shown in a 70-year-old woman presenting with anosmia. Contrast-enhanced computed tomography (CT) images showed ONB at the superior part of both nasal cavities with bilateral ethmoid sinuses and intracranial extension **(A-B)**. Brain CT performed 4 years prior due to headache **(C-D)** showed soft tissue in the slightly expanded left olfactory recess, most likely representing a small ONB that went unrecognized at that time.

Fifty patients (86.2%) had the epicenter of the mass in the superior part of the nasal cavity. One patient in this group had a CT scan performed 4 years prior due to headache (Figure 1).

**Figure 2:**
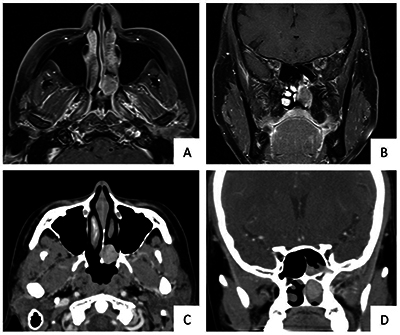
Atypical location of olfactory neuroblastoma in a 36-year-old woman presenting with nasal obstruction for 6 months. Contrast-enhanced T1-weighted imaging with fat suppression **(A-B)** and contrast-enhanced computed tomography **(C-D)** showed an enhanced mass with an epicenter at the middle part of left nasal cavity.

**Figure 3:**
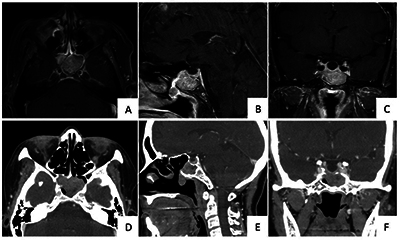
Atypical location of olfactory neuroblastoma in a 63-year-old woman presenting with epistaxis for 5 days. Contrast-enhanced T1-weighted imaging with fat suppression **(A-C)** and contrast-enhanced computed tomography **(D-F)** showed an enhanced mass within both sphenoid sinuses, slightly invading the sellar turcica.

Eight patients (13.8%) had tumors at other atypical locations, seven patients (12.1%) had in the middle part of the nasal cavities (Figure 2), and one patient (1.7%) had in both sphenoid sinuses invading the sellar region (Figure 3).

## Discussion

ONB can affect all age groups, with some studies reporting a bimodal age distribution peaking between 11 and 20 years and later from 41 to 60 years of age [13, 14], while others reporting a single peak in the fifth or sixth decade [13, 15]. Our patient group had ages ranging from 23 to 84 years (mean, 50.6 years), which is consistent with the results of other studies.

There is a lack of consensus on the incidence in both sexes. Some studies have reported that ONB affects men and women equally [16, 17], while a few studies have reported that it is slightly more common in men [18–21]. Our study showed a male predominance (60.3% of patients were men).

Similar to previous reports [6, 18, 19], the two most common presenting symptoms were nasal obstruction (43.1%) and epistaxis (32.8%). Anosmia, rhinorrhea, and eye symptoms (proptosis and eye pain) are occasionally found (3.4%–8.6%).

Similar to other studies [5, 6], ONB typically reveals expansile and destructive growth patterns, invading the adjacent paranasal sinuses in most cases. Intracranial extension was commonly found in 44 patients (75.9%), and intraorbital extension was commonly found in 26 patients (44.8%). In this study, the involvement of both cavities was equally evident without side predominance.

Our study indicated that the upper part of the nasal cavity was the most common location for ONB, accounting for up to 86.2% of cases. However, atypical locations in the middle part of the nasal cavity were identified in 12.1% of cases and 1.7% in the sphenoid sinus, bringing the overall incidence of atypical ONB locations to 13.8%. This could be due to the larger number of participants included in this study when compared to those included in previous studies [5, 6]. To our knowledge, this study included the largest sample population.

The ectopic location of ONB remains speculative but may be explained by ectopic cell rests during embryologic development [4, 7, 12]. In 1971, Jakumeit et al. proposed the first explanation of the origin of ONB, and they supported the concept of ectopic ONB [22]. Embryologically, the olfactory placodes can be divided into two systems. The first system contains the olfactory and vomeronasal nerves, whereas the second system contains the terminal nerve that develops immediately caudal to the first. The terminal nerve ganglion and neurons are distributed across the nasal septum, nasal mucosa, Bowman’s glands, and hypothalamus in a widespread manner. Both the first and second systems degenerate during fetal life; however, the persistence of these cells may be a source for ectopic ONB. Zappia et al. also supported the theory of ectopic cell rests, attributing it to a defect in the embryological migration of neurons from the olfactory placode. They explained the origin of the tumor in the case of Kallman syndrome, a congenital condition with an absence of olfactory bulbs and pituitary hypoplasia using this theory [23].

Imaging has been the mainstream method used for the evaluation and diagnosis of ONB. The location of the nasal mass is an important indicator in the differential diagnosis. Radiologists usually include ONB in the differential diagnosis when the epicenter of the tumor is within the superior part of the nasal cavity; however, it is rarely considered when the center of the lesion is in the rest of the nasal cavity or another location in the head and neck region. Our study highlighted that ONB is not always present in the superior nasal cavity and that radiologists should take this into account.

Distant metastases are rare and have been reported to occur in approximately 12% of ONBs over the course of their natural history, which may appear more than a decade after diagnosis [24]. They often involve the lungs, bones, and, rarely, the liver and mediastinum.

Regional metastases to cervical nodes are relatively common (up to 29%), and the typical spreading to level II nodes with frequent involvement of level I, III, and retropharyngeal nodes [25]. A few nodal metastases are also suggested in our atypical cases, similar to the aforementioned report.

Our study had one main limitation. This was a multicenter study that collected information from three institutes, and some of the patients were referred from other hospitals. Therefore, the images were obtained from different scanners using different protocols. However, the tumor location could still be properly evaluated in all cases.

## Conclusion

ONB is not always present in the upper part or the roof of the nasal cavity, and a significant number of ONBs are occasionally found in the rest of the nasal cavity and other atypical locations.
